# Supplementary material for: Nanoscale Vacuum Diode Based on Thermionic Emission for High Temperature Operation
Source: Micromachines (Basel). 2021 Jun 22;12(7):729. doi: 10.3390/mi12070729 (PMC8307793; doi:10.3390/mi12070729)
Supplement: Supplementary file 1 [file micromachines-12-00729-s001.zip › micromachines-1235268-supplementary.pdf]

micromachines

Supplementary Materials

Nanoscale Vacuum Diode Based on Thermionic Emission for High Temperature Operation

Zhihua Shen [1,*], Qiaoning Li [1], Xiao Wang [2,*], Jinshou Tian [3] and Shengli Wu [4]

[1] School of Electronics and Information Engineering, Nantong Vocational University, Nantong 226007, China; 9000082@mail.ntvu.edu.cn
[2] School of Electronic Information and Artificial Intelligence, Shaanxi University of Science and Technology, Xi'an 710049, China; wangxiao@sust.edu.cn
[3] State Key Laboratory of Transient Optics and Photonics, Xi'an Institute of Optics and Precision Mechanics of CAS, Xi'an, 710119, China; tianjs@opt.ac.cn
[4] Key Laboratory for Physical Electronics and Devices of the Ministry of Education, Xi'an Jiaotong University, Xi'an, 710049, China; slwu@mail.xjtu.edu.cn
* Correspondence: shenzh@mail.ntvu.edu.cn (Z.S.); wangxiao@sust.edu.cn (X.W.)

Citation: Shen, Z.; Li, Q.; Wang, X.; Tian, J.; Wu, S. Nanoscale Vacuum Diode Based on Thermionic Emission for High Temperature Operation. *Micromachines* **2021**, *12*, 729. https://doi.org/10.3390/mi12070729

Academic Editors: Jinn-Kong Sheu and Hieu Pham Trung Nguyen

Received: 10 May 2021
Accepted: 15 June 2021
Published: 22 June 2021

Publisher's Note: MDPI stays neutral with regard to jurisdictional claims in published maps and institutional affiliations.

Copyright: © 2021 by the authors. Licensee MDPI, Basel, Switzerland. This article is an open access article distributed under the terms and conditions of the Creative Commons Attribution (CC BY) license (http://creativecommons.org/licenses/by/4.0/).

The metal-insulator-semiconductor (MIS) structure, with a vacuum channel, was fabricated on a p-type silicon substrate (resistivity = 10 Ωm, (100)-oriented). Si_3N_4, as the insulator layer, was deposited by Plasma-Enhanced Chemical Vapor Deposition (PECVD), and the thickness was approximately 80 nm. Aluminum was deposited by magnetic sputtering on the patterned sample (radius R = 200 μm, thickness = 120 nm) as the cathode. The vacuum channel (radius r = 30 μm) was formed by wet etching with hydrofluoric acid (HF, volume concentration 2%).

As shown in Supplementary Figure S1, the I–U curve of the device was subjected to three different regimes. When the device works in a low voltage range (U < 1 V), the characteristic with a $U^{3/2}$ voltage dependence corresponds to the Child–Langmuir space-charge-limitation (SCL) regime in vacuum, as shown in Supplementary Figure S1a. When the bias is 1 V < U < 14 V, the characteristic corresponds to the Schottky emission regime, as shown in Supplementary Figure S1b. When the bias is U > 14 V (nominal electric field E = U/t > 0.175 V/nm, t is the thickness of insulator), the emission was subjected to F–N regime as shown in Supplementary Figure S1c. Supplementary Figure S1d shows the F–N plot when E > 0.175 V/nm, indicating the field emission essence.

We also measured the I–U curve of the MIS structure without a vacuum channel, as shown in Supplementary Figure S2. It can be seen that the current remains at 1.5 pA when E = 0.18 V/nm, which means the electrons do emit from the sidewall of the cathode against the vacuum channel.

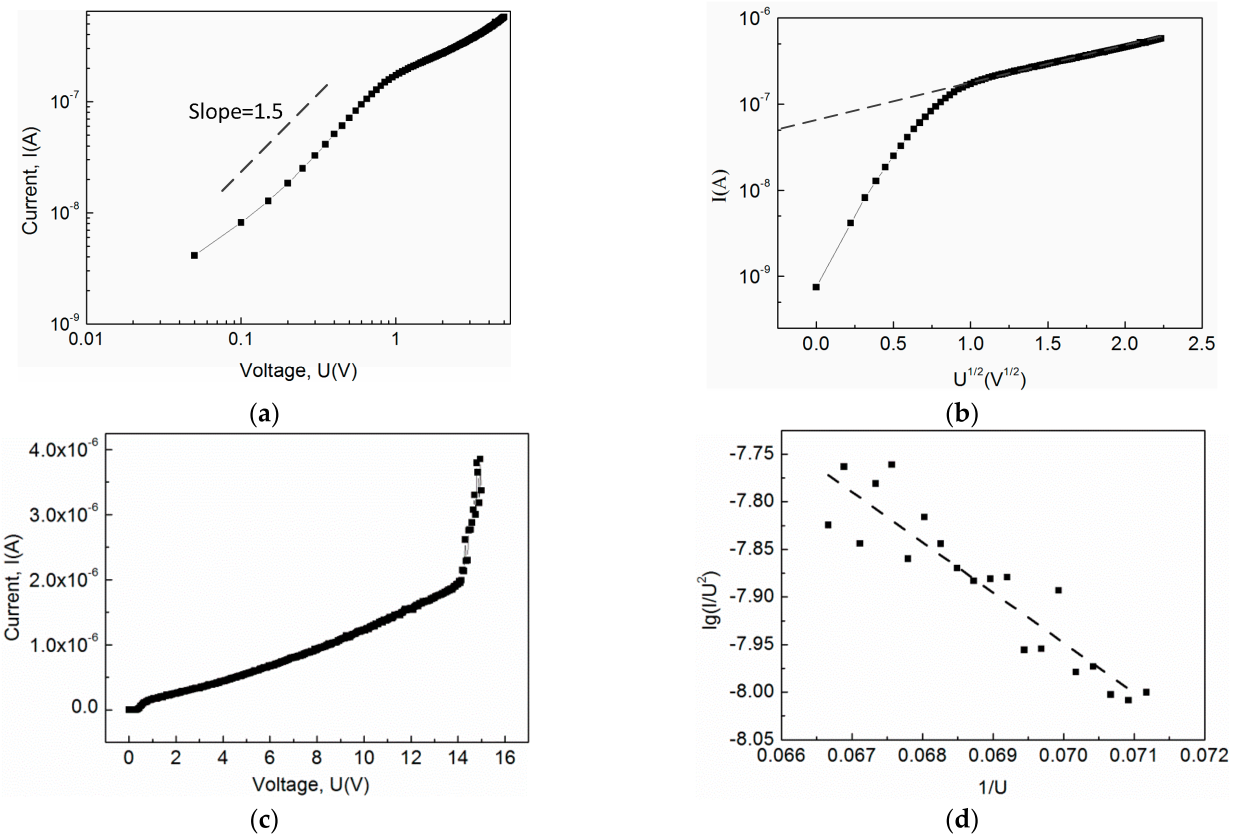

Figure S1. I-U curves of the MIS structure with a vacuum channel. (**a**) Child–Langmuir space-charge-limitation (SCL) regime (U < 1 V) (**b**) Schottky emission regime (1 V < U < 14 V) (**c**) F–N field emission regime (U > 14 V) (**d**)F–N plot (U > 14 V).

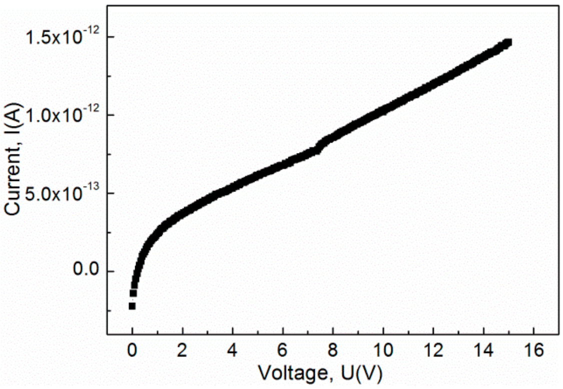

Figure S2. I-U curve of the MIS structure without vacuum channel.
